# “Epidemic” of violence in Brazilian schools and its impact on the health of survivors: a perspective based on adverse childhood experiences

**DOI:** 10.1590/0102-311XEN169723

**Published:** 2024-03-11

**Authors:** Lucas Alves Jural, Patricia de Andrade Risso, Antônio José Ledo Alves da Cunha, Fábio Anevan Fagundes, Andréa Fonseca-Gonçalves, Saul Martins Paiva, Lucianne Cople Maia

**Affiliations:** 1 Faculdade de Odontologia, Universidade Federal do Rio de Janeiro, Rio de Janeiro, Brasil.; 2 Faculdade de Medicina, Universidade Federal do Rio de Janeiro, Rio de Janeiro, Brasil.; 3 Faculdade de Odontologia, Universidade Federal de Minas Gerais, Belo Horizonte, Brasil.

## Introduction

Sequential cases of violent attacks in Brazilian daycare centers, preschools, and schools in the first quarter of 2023 caused the national and international media to advertise an “epidemic” of violence in schools ^1^.

Between 2002 and 2022, 16 violent attacks with fatal victims were reported in schools in Brazil ^2^. In 2023, new attacks occurred, affecting the lives of education professionals, students, and their families, including the “Blumenau Massacre” in a daycare center in Santa Catarina State, when four children aged 4 to 7 years died ^3^.

In addition to violent attacks by aggressors from outside the school, violent actions by students against teachers ^4^ and by teachers against students have also been reported recently in the country, such as the case in which a childcare center director was reported for acts of torture against a 5-year-old child in São Paulo ^5^.

For the World Health Organization (WHO), violence is the intentional use of physical force or power, threatened or actual, against oneself, another person, or against a group or community, that either results in or has a high likelihood of resulting in injury, death, psychological harm, maldevelopment, or deprivation ^6^ of a physical, psychological, or sexual nature, or through negligence and abandonment ^6^ ^,^ ^7^. From the perspective of students as victims, these violent events constitute explicit forms of adverse childhood experiences, which can cause irreversible impacts on their lives ^8^ ^,^ ^9^ ^,^ ^10^.

Despite the spread of news regarding the attacks followed by physical injuries and/or deaths in Brazilian schools, little is discussed about the effects these episodes may have on the lives and health of schoolchildren who suffered or witnessed these attacks. Using adverse childhood experiences concepts, this study aims to discuss the impact of violent attacks in schools on the health of surviving students and reflect on preventive approaches for these events.

## Adverse childhood experiences and the impact on health over the years

Adverse childhood experiences refer to traumatic events or situations a person experienced during childhood and adolescence. Brazilian studies have reported a high prevalence of at least one adverse childhood experience among children, adolescents, and young adults ^11^ ^,^ ^12^. These traumatic events in an individual’s childhood and adolescence include bullying and violence within the community - where school is inserted ^13^.

In addition to being associated with early mortality and an increase in chronic diseases ^14^, adverse childhood experiences can lead to toxic stress and mental health problems, impairing the development of victims, from childhood to adulthood. Depending on the magnitude of the event, adverse childhood experiences can help trigger depression and suicidal ideations among adolescents and adults ^15^.

Adverse childhood experiences have several consequences on the health of survivors of violent events and can generate unhealthy habits and aggravate other pathological processes ^16^ ^,^ ^17^. Associations are also observed between adverse childhood experiences and attention deficit hyperactivity disorder in children and adolescents, also when community violence was investigated separately ^18^. Also, children who had traumatic events from 4 to 5 years of age are more likely to develop behavioral and developmental disorders ^19^, serving as a warning, especially in cases such as the Blumenau Massacre.

Reintegration of victims into the school environment should be widely discussed, considering the strong effect on physical and mental health and academic performance ^20^. Despite the scarcity of studies investigating adverse childhood experiences in schools and, in particular, adverse childhood experiences involving violent events, the provision of medical and psychological support should be considered, with pedagogical adaptations and changes in the physical area of the school. Also, healthy home environment and community can also avoid problems in school performance, as these are protective factors for children who experience adverse childhood experiences ^20^. This would create a support network working toward giving a new meaning to the environment where children, adolescents, and teachers had experienced violence.

## Prevention strategies based on the multidimensional origin of attacks

Identifying the origins and determinants of violent attacks in schools is a big challenge, especially when observing the multiple types of aggressors in the cases mentioned above, i.e., external agents ^3^, students ^4^, and teachers ^5^. In view of these facts, reflecting, proposing, and adopting measures to prevent and mitigate this “epidemic” involves public policies for the creation of multiprofessional centers to understand and act on the root of such violence, as well as providing continuous training for professionals of the education system (including teachers, directors, inspectors, and others) on how to apply preventive approaches and identify and reduce damage caused by violence in school in its different forms, including violence of smaller magnitude.

Several authors have analyzed school violence based on Bronfenbrenner’s bioecological theory, considering the influences of the environment, interpersonal relationships, and social factors to which an individual is exposed, and how these aspects can increase the risk of victimization or perpetration of violence ^8^ ^,^ ^20^. In this model, violence develops from ecological systems that act simultaneously ^8^, including home, school, community environments, considering that interactions in these environments can impact one’s development. Therefore, healthier and more favorable ecosystems contribute to better development, while more adverse and dysfunctional environments lead to higher chances of violent and harmful behavior for oneself and others.

In response to the attacks, the Executive Branch in Brazil announced some measures to reduce the cases of violence in schools, including the creation of a monitoring system with a hotline so people can report cases of school violence and incentive toward investigations and epidemiological studies based on these events ^21^. Also, an amendment to *Law n. 8,072*, of July 25, 1990, which addresses heinous crimes, has been proposed to include cases of very serious violence with physical injury followed by death occurring in schools ^22^.

Given the complexity of the problem, a broader discussion is required, promoting reflections on the school space and how it is inserted in the aggressor’s ecosystem, as well as the reasons for making it a place of violence in the present or future - in the present, as in the case of students and teachers who committed violent acts in the school due to their school experiences as a student or worker, and in the future, when a violent attack is potentially associated with prior experiences, as in the Realengo Massacre in 2011, when a former student of a municipal school in Rio de Janeiro invaded and murdered 12 teenagers. As explained by the aggressor’s family and close friends, he was a victim of bullying and physical attacks at school ten years before the attack ^23^. According to an American study, in shooting attacks in schools, 72% of the aggressors had at least one adverse childhood experience, such as exposure to violence, and 60% were victims of bullying ^24^, highlighting that such violent acts may be connected to the accumulation of experiences in the childhood.

## Final considerations

Despite the growing number of evidence about the cumulative impact of adverse childhood experiences on the health of individuals, emphasis should be placed on the production of scientific knowledge about specific impacts of violent events in the school environment, especially when considering the important role of school in the child and adolescent development.

In this perspective, schools can influence the behavior of students and other actors using different approaches, including the identification of vulnerable children/adolescents or chronically exposed to risk situations, violence, bullying, and mistreatment. Also, schools can encourage the exchange of information, inspiring students to talk and think about their own experiences, and can identify and refer students to health services, and develop health promotion actions. It is also important to highlight the role of state and municipal departments, in particular health and education departments, in providing continuing training for professionals involved in this process as well as human and financial resources for the implementation of articulated measures. These strategies may involve, for example, practices that simulate real cases frequently experienced in everyday life in school and how to handle these cases, aiming to ensure the integrity of all actors involved in the educational complex. Reflection on the culture of school violence should not focus exclusively on aggressors and victims, but also include everyone that is vulnerable and at risk, teaching people how to recognize, report, and not feel intimidated. Home, community, and school must be positive environments so students can feel safe.

Understanding the etiological diversity and ecological development of violence becomes a *sine qua non* condition to avoid, in the long run, new cases of the “violence pandemic” in schools. Moreover, primary health care in or out of schools should include screening for adverse childhood experiences ^25^ to protect children and adolescents and support the development and strengthening of adverse childhood experiences monitoring, contingency, and prevention programs for schoolchildren, addressing adverse childhood experiences that occur in the school environment or not, since social, interpersonal, and environmental relationships can also occur in this space and, consequently, encourage the development of violent attitudes and its outcomes.

Finally, it is necessary to address the challenge of reinserting children and adolescents who survived these attacks into the school environment, understanding the potential impact on their overall development over the years. In addition to the multiprofessional support mentioned above, joint strategies must be developed that show the real meaning of school to these individuals.
